# Hand Hygiene Knowledge and Perception Survey for Healthcare Workers in Government Hospitals (GHs) in Bahrain

**DOI:** 10.7759/cureus.50126

**Published:** 2023-12-07

**Authors:** Saleh F Sowar, Rommel Acunin, Harold C Cabanalan, Tamer T Abo Arisheh, Safa Alkhawaja

**Affiliations:** 1 Infection Prevention and Control, Government Hospitals, Manama, BHR; 2 Medicine, Salmaniya Medical Complex, Manama, BHR

**Keywords:** healthcare workers, perception, knowledge, bahrain, hand hygiene, who guidelines, healthcare worker perception, knowledge assessment, infection prevention and control, hand hygiene compliance

## Abstract

Introduction and aim

Healthcare-associated infections (HAIs) are a serious concern in all healthcare facilities as they may lead to many serious consequences, like prolonged hospitalization, increased mortality and morbidity, and extra costs. Effective hand hygiene (HH) is the primary proven measure known to be effective in reducing the risk of HAIs in all healthcare settings. This study aimed to measure the healthcare workers' (HCWs) knowledge and perception of HH at government hospitals (GHs) in Bahrain.

Subjects and methods

This cross-sectional study was conducted among HCWs working in GHs in the Kingdom of Bahrain. A self-administered questionnaire was distributed among the targeted population using a Google survey (Google LLC, California, USA). The questionnaire includes socio-demographic data (e.g., age, gender, nationality, etc.) and a validated WHO questionnaire measuring perceptions and knowledge toward HH.

Results

Of the 285 HCWs, 75.4% were females, and 48.1% were aged between 31 and 40 years old. The overall mean perception was 35.9 (SD 6.93) out of 42 points, with nearly 80% of HCWs considered to have good perception levels. The overall mean knowledge score was 11.4 (SD 1.37) out of 14 points. Accordingly, 75.4% were considered to have good knowledge levels. Factors associated with increased perception include increasing age, female gender, non-Bahraini, being a nurse with increasing years of working experience, and receiving formal training in HH. Being a nurse was the sole significant predictor of increased knowledge.

Conclusion

The knowledge and perception of HH among HCWs were sufficient. Being a nurse was a significant predictor of both knowledge and perception. Further, a significant positive correlation was noted between the knowledge and perception scores. Maintaining the level of knowledge and perception of HH could lead to improved HH compliance among HCWs.

## Introduction

Healthcare-associated infections (HAIs) are a serious concern in all healthcare facilities, as they may lead to many serious consequences, like prolonged hospitalization, increased mortality and morbidity, and extra costs. Most HAIs can be transmitted from patient to patient via the hands of healthcare workers (HCWs). Therefore, poor compliance with hand hygiene (HH) among the healthcare team can be considered a common cause of the transmission of HAIs in healthcare facilities [1].

HH is defined as hand cleansing by using water, detergent, and/or alcohol-based hand sanitizers to remove transients from hands [2]. Effective HH is the primary proven measure known to be effective in reducing the risk of HAIs in all healthcare settings. Unfortunately, the prevalence of HAIs continues to rise, and it is estimated that, annually, millions of patients suffer from HAIs globally. Therefore, compliance with infection control measures is necessary to reduce HAIs [2].

Though HH is a simple procedure, compliance with HH among HCWs is still under expectations [2,3]. Possible causes of low compliance with HH among HCWs include a lack of HH training, a high workload, and a shortage of HH supplies. To address this problem, the concept of "my five moments for hand hygiene" was introduced by the World Health Organization (WHO) in 2005. These five moments that call for using HH include the moment before touching a patient, performing aseptic and clean procedures, being at risk of exposure to body fluids, touching a patient, and touching a patient's surroundings. This concept has been used to improve the understanding, training, monitoring, and reporting of HH among HCWs [2].

The risk of HAIs can be reduced by improving HCWs' compliance with HH by following the WHO multimodal HH improvement strategy, which includes providing enough supplies of HH resources and HH reminders at the point of care and providing proper HH education and training to all HCWs with close monitoring and feedback [2,3]. Moreover, HCWs' HH perception and knowledge of recommended HH practices should be considered important factors affecting the HH compliance rate [4,5]. HCWs should have excellent knowledge and a positive attitude toward HH to have high compliance with HH practices as the global infection prevention and control bodies advocate.

Literature review

Numerous studies across the globe evaluated HCWs' knowledge, attitude, and practice of HH. Most of these studies utilize the WHO multimodal HH improvement strategy evaluation and feedback questionnaire as a primary tool for data collection [6]. In the Kingdom of Bahrain, there was one study conducted recently at a prominent university that assessed the knowledge and practice of medical students on HH [7]. Apart from that, no other related research was published in a healthcare setting in the country. Auspiciously, there is plenty of literature available from neighboring countries [8-18]. The majority of these studies were from different cities and provinces of Saudi Arabia [8-15]. In one study done at a national level to assess HCWs' knowledge, attitude, and practice toward HH, around 7,153 healthcare staff participated using a self-administered questionnaire from twenty different health regions in the Kingdom [8]. The study found that the mean HH knowledge percentage score among HCWs was 65.5%. This finding was consistent with the results of studies conducted in Iran and India [17,19]. Similar studies from other Asian and African countries that apply qualitative measurements reported a moderate to good level of knowledge on HH among HCWs [7,9-12,20-24]. On the contrary, one study in Romania reported inadequate knowledge for almost 68% of nurses who participated in the survey [25].

Regarding HH perception, various papers tackled the impact of HCWs' perceptions on HH practices and compliance [9,17,18,24]. In Qassim, Saudi Arabia, research done during the peak of the COVID-19 pandemic reported that HCWs who received appropriate training on HH were more likely to have moderate/good perceptions [9]. Another study from four different tertiary care centers in Malaysia [26] found a significant positive correlation between HH knowledge and HH perceptions. A recent publication from Denmark [27] focusing on perception and self-reported HH compliance among emergency medical services personnel reported that HCWs' perceptions were extensively influenced by several factors, such as support from the management, availability of HH supplies at the point of use, and having a good role model at the workplace. In addition to these factors, having a safety culture, regular training and education, providing reminders at the workplace, and continuous monitoring and feedback are some of the major elements that affect HCWs' knowledge and perceptions toward HH [8,9,11-13,17-20,24-30]. Furthermore, many of these studies suggested that HH knowledge and perceptions increased with HCWs' age and years of experience [10,14,17,18,27]. Hence, this study sought to determine HCWs' knowledge and perception of HH at government hospitals (GHs).

## Materials and methods

This cross-sectional study was conducted among HCWs working in GHs, Bahrain. HCWs aged between 20 and 60 who provided direct contact with patients were eligible to participate. We excluded HCWs over 60 years old, as this is the retirement age for our institution. The sample size was calculated using Raosoft software (Raosoft Inc., Washington, USA), estimating around 5000 overall population size of HCWs in GHs, with a confidence level of 95% and a margin of error of 5%; the estimated sample size was 357. A convenience sampling technique was employed until we achieved the clinical significance number required by the study. Data were collected from September 2023 to November 2023 using an online survey. A self-administered questionnaire was used, comprising four parts: socio-demographics (i.e., age, gender, years in practice, etc.), WHO HH knowledge, and perception questionnaires [2]. Participants were informed of the voluntary nature of this study and provided informed consent before completing the questionnaire. The Ethics Committee of Government Hospitals, Bahrain, approved this research project (approval number: 99260923).

Questionnaire criteria

HCWs' perception of HH has been assessed using a seven-item questionnaire with seven-point Likert scale categories ranging from "not effective" coded with 1 to "very effective" coded with 7. The total perception has been calculated by adding all seven items. A score ranging from 7 to 42 has been generated. The higher the score, the higher the perception of HH. By using 50% and 75% as cutoff points to determine the level of perception, HCWs were considered to have poor perception if the score was below 50%, 50% to 75% were considered moderate, and above 75% were considered to have good perception levels. The reliability of the perception questionnaire has a Cronbach's alpha of 0.885%, or 88.5%, indicating very good internal consistency. Thus, this questionnaire was valid for use in this study.

Likewise, the knowledge of HH has been measured using a 14-item questionnaire, with the correct answer for each question identified and coded with 1, while the incorrect answer has been coded with 0. Adding all 14 items generated a score range of 1 to 14 points. The greater the score, the greater the knowledge about HH. Similar criteria taken from perception have been used to classify the level of knowledge (50% and 75% cutoff points) as poor, moderate, and good knowledge levels.

Statistical analysis

The data analyses were carried out using SPSS Statistics version 26 (IBM Corp. Released 2019. IBM SPSS Statistics for Windows, Version 26.0. Armonk, NY: IBM Corp). Categorical variables were given as numbers and percentages (%), while continuous variables were computed and summarized as the mean and standard deviation. The differences in the knowledge scores of perception and knowledge in relation to HCW's socio-demographic characteristics have been performed using the Mann-Whitney Z-test and Kruskal-Wallis H-test. A post hoc test was also performed to determine the multiple mean differences in perception and knowledge scores in relation to job category. The normality test was carried out using the Shapiro-Wilk test and the Kolmogorov-Smirnov test. The knowledge and perception scores follow a non-normal distribution. Therefore, the non-parametric tests were applied. In addition, the Spearman correlation coefficient was also used to determine the correlation between perception and knowledge scores. Statistical significance has been set to p<0.05.

## Results

We distributed 357 questionnaires to HCWs, and 285 were returned, giving an overall response rate of 79.8%. Table 1 presents the socio-demographic characteristics of the HCWs. The most common age group was 31 to 40 years old (48.1%), with females being dominant (75.4%). More than half (50.5%) were Bahrainis, and approximately three-quarters (75.4%) were nurses. HCWs who were working in the medical department constitute 45.3%. With respect to years of working experience, 27.4% had working experience between 11 and 15 years. The proportion of HCWs who received formal training in HH was 83.9%, while those who routinely use an alcohol-based hand rub for HH were 96.1%.

**Table 1:** Socio-demographic characteristics of HCWs (n=285) HCWs: healthcare workers

Study data	N (%)
Age group	
20-30 years	52 (18.2%)
31-40 years	137 (48.1%)
41-50 years	67 (23.5%)
51-60 years	23 (08.1%)
>60 years	06 (02.1%)
Gender	
Male	70 (24.6%)
Female	215 (75.4%)
Nationality	
Bahraini	144 (50.5%)
Non-Bahraini	141 (49.5%)
Job category	
Nurse	212 (74.4%)
Physician	41 (14.4%)
Other allied HCWs	32 (11.2%)
Department	
Medical department	129 (45.3%)
Surgical department	88 (30.9%)
Excellency centers	45 (15.8%)
Radiology	03 (01.1%)
Physiotherapy	02 (0.70%)
Lab	14 (04.9%)
Respiratory therapy	04 (01.4%)
Years of working experience	
<1 year	05 (01.8%)
1-5 years	54 (18.9%)
6-10 years	50 (17.5%)
11-15 years	78 (27.4%)
16-20 years	56 (19.6%)
>20 years	42 (14.7%)
Did you receive formal training in HH in the last three years?	
Yes	239 (83.9%)
No	46 (16.1%)
Do you routinely use an alcohol-based hand rub for HH?	
Yes	274 (96.1%)
No	11 (03.9%)

**Figure 1:**
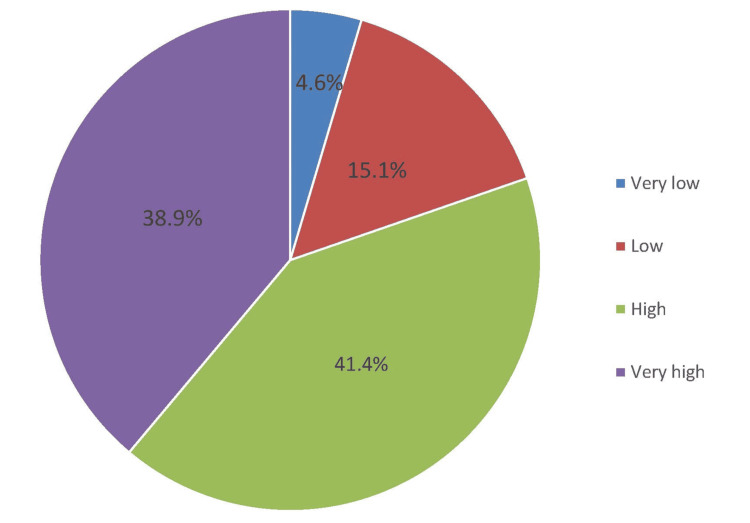
Perceived impact of a HAI on a patient's clinical outcome

In Figure 1, the perceived impact of a HAI on patients was high among 41.4% of HCWs, followed by very high (38.9%).

**Figure 2:**
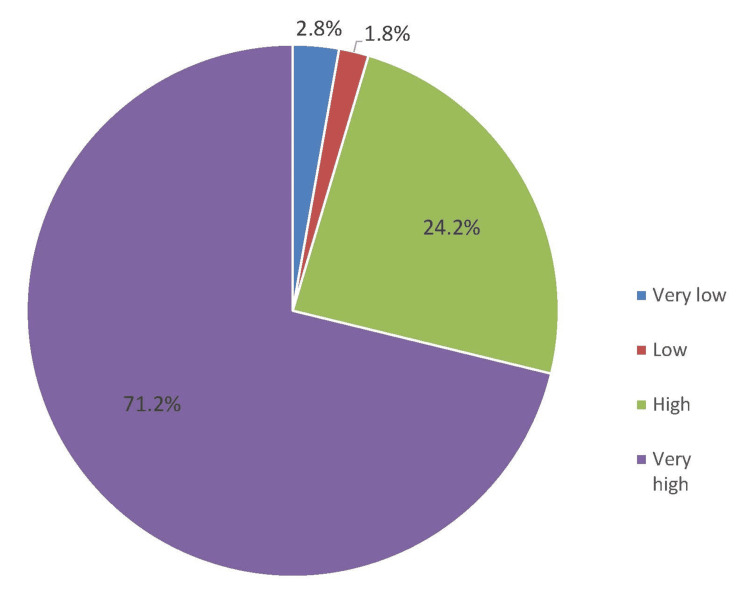
Perceived effectiveness of HH in preventing HAI

In Figure 2, the perceived effectiveness of HH in preventing HAI was very high among 71.2% of HCWs, followed by high (24.2%).

In Table 2, HCWs believed that HH would be very effective if the reminders for HH compliance were done through displayed posters (mean score: 6.12), followed by HH education (mean score: 6.09) and the availability of alcohol-based hand rub at each point of care (mean score: 6.05). The overall mean perception score was 35.9 (SD 6.93), with poor, moderate, and good perception levels constituting 4.2%, 16.1%, and 79.6%, respectively.

**Table 2:** Assessment of perception of HH (n=285) Responses range from "not effective" coded with 1 to "very effective" coded with 7 HH: hand hygiene, GHs: government hospitals, HCW: healthcare worker

Statement	Mean ± SD
HH posters are displayed at the point of care as reminders	6.12 ± 1.31
Each HCW receives education on HH	6.09 ± 1.47
The GHs make alcohol-based hand rub always available at each point of care	6.05 ± 1.39
What importance does the head of your department attach to the fact that you perform optimal HH?	5.95 ± 1.38
Leaders and senior managers at GHs support and openly promote HH	5.94 ± 1.43
HCW regularly receives feedback on their HH performance	5.70 ± 1.69
Total perception score	35.9 ± 6.93
Level of perception	
Poor	12 (04.2%)
Moderate	46 (16.1%)
Good	227 (79.6%)

In the HH knowledge assessment (Table 3), we noticed that most of our HCWs demonstrated good knowledge of HH basic facts. Most notably about the action to prevent transmission of germs before touching the patients (correct answer: 98.6%) or after touching a patient (correct answer: 97.5%), avoidance of harmful germs that may increase the likelihood of colonization of hands (correct answer: 96.5%), and after exposure to the immediate surroundings of a patient (correct answer: 96.8%). However, we also noted gaps in HH knowledge, including that hand rubbing with an alcohol-based rub is more effective than handwashing with soap (correct answer: 37.9%), and hand rubbing is necessary before giving an injection (correct answer: 37.5%). The overall mean knowledge score was 11.4 (SD 1.37). Accordingly, moderate and good knowledge levels were found in 24.6% and 75.4%, respectively. None of the HCWs were classified as having poor knowledge levels.

**Table 3:** Assessment of knowledge toward HH (n=285) HH: hand hygiene, HCWs: healthcare workers

Statement	N (%)
Which of the following is the main route of cross-transmission of potentially harmful germs between patients in a healthcare facility? (HCWs' hands when not clean)	198 (69.5%)
Which of the following is considered proper HH? (Hand wash with water and soap for 30-60 seconds or hand rub with alcohol-based rub for 20-30 seconds)	258 (90.5%)
Which of the following HH actions prevents transmission of germs to the patient?	
Before touching a patient (yes)	281 (98.6%)
Immediately before a clean/aseptic procedure (yes)	274 (96.1%)
Which of the following HH actions prevents transmission of germs to the HCW?	
After touching a patient (yes)	278 (97.5%)
Immediately after a risk of body fluid exposure (yes)	278 (97.5%)
After exposure to the immediate surroundings of a patient (yes)	276 (96.8%)
To reduce the risk of cross-infection, hand rubbing with an alcohol-based rub is more effective than handwashing with soap (true)	108 (37.9%)
What is the minimal time needed for alcohol-based hand rub to kill most germs on your hands? (20 seconds)	232 (81.4%)
Which type of HH method is required in the following situations?	
Before palpation of the abdomen (hand rub)	201 (70.5%)
Before giving an injection (hand rub)	107 (37.5%)
After visible exposure to blood (hand rub)	269 (94.4%)
After removing used gloves (hand rub)	207 (72.6%)
Which of the following should be avoided, as associated with increased likelihood of colonization of hands with harmful germs? (All of the above)	275 (96.5%)
Total knowledge score (mean ± SD)	11.4 ± 1.37
Level of knowledge	
Poor	0
Moderate	70 (24.6%)
Good	215 (75.4%)

**Figure 3:**
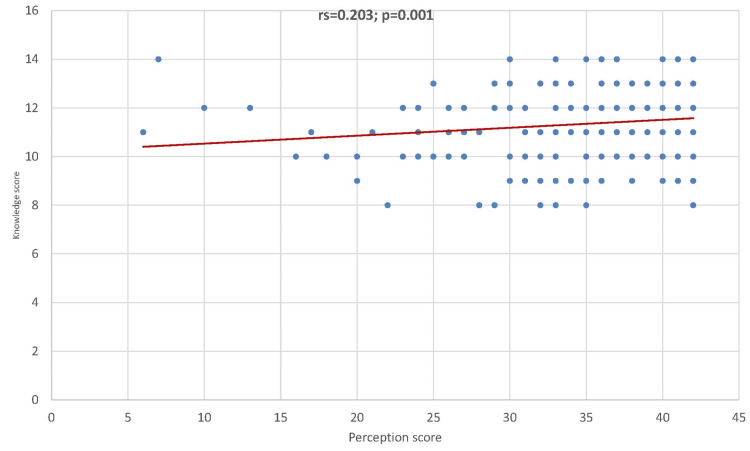
Correlation between perception score and knowledge score

In Figure 3, the Spearman correlation coefficient indicated that the correlation between perception score and knowledge score was positively statistically significant (rs=203; p=0.001).

When measuring the differences in the score of perception and knowledge in relation to the socio-demographic characteristics of the HCWs (Table 4), it was observed that a higher perception score was more associated with being older (Z=3.090; p=0.002), being female (Z=1.974; p=0.048), non-Bahraini (Z=5.250; p<0.001), being a nurse (H=25.303; p<0.001), increasing years of working experience (Z=3.312; p=0.001), and receiving formal training in HH (Z=2.982; p=0.003). On the other hand, a higher knowledge score was more associated with being a nurse (H=9.008; p=0.011).

**Table 4:** Differences in the scores of perception and knowledge according to the socio-demographic characteristics of HCWs (n=285) a p-value has been calculated using the Mann-Whitney Z-test b p-value has been calculated using the Kruskal-Wallis H-test ** Significant at p<0.05 level

Factor	Perception score (42) mean ± SD	Z/H-test; p-value	Knowledge score (14) mean ± SD	Z/H-test; p-value
Age group^a^				
≤40 years	35.1 ± 6.89	3.090; 0.002**	11.4 ± 1.32	0.544; 0.587
>40 years	37.1 ± 6.97		11.3 ± 1.53	
Gender^a^				
Male	34.8 ± 6.66	1.974; 0.048**	11.1 ± 1.61	1.693; 0.090
Female	36.2 ± 7.00		11.5 ± 1.28	
Nationality^a^				
Bahraini	33.9 ± 7.21	5.250; <0.001**	11.3 ± 1.36	1.469; 0.142
Non-Bahraini	37.8 ± 6.07		11.5 ± 1.39	
Job category^b^				
Nurse	36.9 ± 6.43	25.303; <0.001**	11.5 ± 1.32	9.008; 0.011 **
Physician	31.6 ± 8.04		10.9 ± 1.46	
Other allied HCWs	33.9 ± 6.28		11.0 ± 1.46	
Department^b^				
Medical department	35.9 ± 6.97	1.406; 0.704	11.5 ± 1.47	1.678; 0.642
Surgical department	35.8 ± 7.57		11.3 ± 1.31	
Excellency centers	36.4 ± 5.68		11.4 ± 1.25	
Other departments	34.6 ± 6.65		11.3 ± 1.36	
Years of working experience^a^				
≤10 years	34.2 ± 7.44	3.312; 0.001**	11.4 ± 1.24	0.191; 0.849
>10 years	36.9 ± 6.41		11.4 ± 1. 45	
Received formal training in HH in the last three years^a^				
Yes	35.6 ± 6.32	2.982; 0.003**	11.4 ± 1.35	1.634; 0.102
No	32.4 ± 8.81		11.1 ± 1.45	
Routinely use an alcohol-based hand rub for HH^a^				
Yes	35.9 ± 6.89	0.377; 0.706	11.4 ± 1.38	0.998; 0.318
No	34.3 ± 8.22		11.0 ± 1.09	

In post hoc analysis (Table 5), it was observed that the perception score was statistically significant between physician and nurse (p<0.001), while the knowledge score was also statistically significant between nurse and physician (p=0.033).

**Table 5:** Multiple mean differences in the scores of perception and knowledge in relation to job categories (n=285) Post hoc analysis was conducted using the Dunn-Bonferroni test * The mean difference is significant at the 0.05 level

Dependent variable	(I) Job category	(J) Job category	Mean difference (I-J)	Std. error	Sig.	95% confidence interval	
						Lower Bound	Upper Bound
Total perception score	Nurse	Physician	5.38162^*^	1.13708	0.000	2.6431	8.1201
		Other allied HCWs	3.02948	1.26399	0.052	-0.0146	6.0736
	Physician	Nurse	-5.38162^*^	1.13708	0.000	-8.1201	-2.6431
		Other allied HCWs	-2.35213	1.57212	0.407	-6.1384	1.4341
	Other allied HCWs	Nurse	-3.02948	1.26399	0.052	-6.0736	0.0146
		Physician	2.35213	1.57212	0.407	-1.4341	6.1384
Total knowledge score	Nurse	Physician	0.59204^*^	0.23161	0.033	0.0342	1.1498
		Other allied HCWs	0.51887	0.25746	0.134	-0.1012	1.1389
	Physician	Nurse	-0.59204^*^	0.23161	0.033	-1.1498	-0.0342
		Other allied HCWs	-0.07317	0.32022	1.000	-0.8444	0.6980
	Other allied HCWs	Nurse	-0.51887	0.25746	0.134	-1.1389	0.1012
		Physician	0.07317	0.32022	1.000	-0.6980	0.8444

## Discussion

The present study investigated HCWs' knowledge and perceptions of HH among GHs. There were limited studies conducted in Bahrain that measured HH familiarity and understanding. Although one study evaluated HH knowledge and practice among medical students, none of the literature was from Bahrain HCWs. Thus, this study was the first in Bahrain to quantify HH knowledge of HCWs, which could support the literature on the ongoing discussion about HCWs' HH awareness and compliance, considering that GHs are the biggest hospitals in Bahrain, consisting of approximately 1200 beds. Hence, the HH knowledge and compliance of our HCWs are crucial to our institution.

Knowledge of HH

According to our results, HCWs' knowledge of HH is adequate. Based on the given criteria, approximately three-quarters (75.4%) were considered to have good knowledge levels. The rest were considered to have moderate knowledge levels (24.6%). Interestingly, none of the HCWs were categorized as having a poor level of knowledge (mean score: 11.4 out of 14 points). This is consistent with the reports of Agbana et al. [23] and Bader Aldeen and Kheder [24]. Both pieces of literature documented a high level of knowledge among HCWs, with 98.9% and 99.3%, respectively. Several studies documented a moderate level of knowledge among HCWs [7-10,18]. In contrast, a study done in Romania [25] found inadequate knowledge among nurses, compromising 68%, which was lower than our report. One of the main reasons why our HCWs demonstrated a high level of knowledge was that this survey was done during the post-pandemic era, wherein compliance with HH had been previously discussed across the literature, and tremendous efforts had been made globally to increase awareness about HH compliance.

A significant factor of knowledge

Data from our study indicates that nurses were more likely to exhibit better HH understanding than the other HCWs. This is contrary to the report of Ng et al. [16], who reported that doctors' HH knowledge was slightly better than that of nurses. In Qassim, Saudi Arabia [9], male HCWs working in private hospitals were less associated with having moderate or good HH knowledge than females. Similarly, in Najran, Saudi Arabia [10], being older, female, working in a surgery department, having more than 10 years of experience, living in a shared accommodation, and having associated chronic disease tended to make people more knowledgeable about HH, while in Pakistan [22], based on multivariate regression estimates, only work experience and previous education were determined to be the most important predictors of HH knowledge. However, in our study, we found no significant differences between knowledge scores in relation to demographic variables, including age, gender, nationality, department, and years of working experience (p>0.05), which was consistent with the report of Alhraiwil et al. [8].

Specific details of knowledge

Regarding the specific details of knowledge, our HCWs showed excellent knowledge, as more than 90% were able to identify the correct knowledge answer, particularly in the proper HH, preventive action toward transmitting germs to patients or co-workers, HH methods after visible exposure to blood, and avoidance of the harmful germ that increased the likelihood of colonization of hands. However, despite this scenario, some gaps in knowledge have to be addressed, including a lack of understanding about the proper HH before giving an injection (correct answer: 37.5%) and the effectiveness of hand rubbing with an alcohol-based rub rather than handwashing with soap (correct answer: 37.9%). In a survey conducted by Mujbel et al. [7], medical students were seen to have a lack of knowledge about the prevention of transmission of germs to patients (27%) or HCWs (27%). They seem to have a misconception about the importance of hand rubbing before palpation of the abdomen (34%), before giving an injection (34%), after making a patient bed (31.4%), and after removing examination gloves (28.9%). This is comparable to the study done in the Aseer Region, Saudi Arabia [11], as only 26.4% of the HCWs were aware that existing germs in patients were the most frequent source of pathogens in a healthcare facility, and only 54.8% achieved proper knowledge about the minimum time needed for an alcohol-based hand rub to destroy most germs on hands.

Perception

The perception of our HCWs regarding HH mirrored the results of our knowledge. According to our results, nearly 80% of our respondents were considered to have a good level of perception. Only 4.2% were categorized as poor, while 16.1% were considered moderate (mean score: 35.9 out of 42 points). Among Iranian nurses [18], approximately 65.4% demonstrated high HH perception, while in Sudan [24], good and fair perceptions were found in 45.2% and 54.8%, respectively. The deficiency of information about HH might negatively affect perception, leading to decreased HH compliance. Hence, constant monitoring of HH is essential to maintaining a safe hospital environment.

A significant factor in perception

This study identified several demographic variables that positively influence perception, including older age, female gender, non-Bahraini, being a nurse, and increasing years of working experience. In the Qassim Region, Saudi Arabia [9], HCWs with higher education who were working in private hospitals were less associated with having moderate or good HH perception levels, while in Iran [18], significant predictors of perception include age, working experience, and workplace ward.

Specific details of perception

Pertaining to the specific details of perception, the rating of our HCWs on a six-item perception questionnaire seems to be more than satisfactory. Out of seven points (Likert scale category), the mean score for each item was approximately six points, with the highest rating on the statement "HH posters should be displayed at the point of care reminders" (mean score: 6.12), while the lowest rating was the statement about "HCWs regularly received feedback on their HH performance" (mean score: 5.70). Further, regarding the perceived impact of HAI on patients, most of our HCWs believed that the impact was high (41.4%) to very high (38.9%). In addition, the perceived effectiveness of HH in preventing HAI was very high, at 71.2% and 24.2%, respectively. In Dawadmi, Saudi Arabia [14], nearly two-thirds (64.3%) of healthcare personnel (HCPs) believed that HAI greatly influences patients' clinical outcomes. This opinion was echoed by the Iran nurses [17], wherein 68.3% of them believed that the effect of HCAI on patients' outcomes was high and very high among 19.8%. It is necessary to identify factors affecting HH perception, which could translate to better HH compliance.

Correlation between knowledge and perception scores

According to our scatter plot, there was a positive but weak correlation between the knowledge score and perception score (p=0.001), suggesting that the increase in the knowledge score is correlated with the increase in perception score. This mirrored the results of the study done by Abd Rahim and Ibrahim [26], employing the Pearson correlation coefficient; they found a significant positive correlation between knowledge and perceptions score (p<0.001).​​​​​​​

Attendance to training and compliance with HH

Most of our respondents had attended training related to HH (83.9%), and this positively influenced perception (p=0.003) but not knowledge (p=0.102). On the other hand, almost all (96.1%) complied with the routine use of alcohol-based hand rubs for HH. However, this did not differ significantly with knowledge and perception (p>0.05). This is consistent with the study of Bader Aldeen and Kheder [24], who reported that formal HH training has no significant relationship with the level of knowledge, and fair perception was greater among HCWs who did not participate in HH training. However, in Vietnam [30], based on the logistics regression model, HCWs who received training and clinical information related to HH were predicted to have better knowledge of HH.

## Conclusions

There was adequate knowledge and perception of HCWs regarding hand hygiene. Nurses seem to have a better understanding of HH than the others. However, older female HCWs who had more years of experience and attended formal training in HH tended to have better perceptions than any other HCWs. Although there was satisfactory HH knowledge and perception among our HCWs, some gaps needed to be addressed, particularly in HH knowledge. Hence, continuous education is needed to update HCWs' knowledge about HH. Furthermore, multimodal schemes and interventions adapted locally are essential to maintaining hand hygiene compliance.
